# WholeCellViz: data visualization for whole-cell models

**DOI:** 10.1186/1471-2105-14-253

**Published:** 2013-08-21

**Authors:** Ruby Lee, Jonathan R Karr, Markus W Covert

**Affiliations:** 1 Department of Bioengineering, Stanford University, Stanford, CA 94025, USA; 2 Graduate Program in Biophysics, Stanford University, Stanford, CA 94025, USA

**Keywords:** Whole-cell modeling, Data visualization, Cell physiology, Computational biology, Mycoplasma, Bacteria, Systems biology

## Abstract

**Background:**

Whole-cell models promise to accelerate biomedical science and engineering. However, discovering new biology from whole-cell models and other high-throughput technologies requires novel tools for exploring and analyzing complex, high-dimensional data.

**Results:**

We developed WholeCellViz, a web-based software program for visually exploring and analyzing whole-cell simulations. WholeCellViz provides 14 animated visualizations, including metabolic and chromosome maps. These visualizations help researchers analyze model predictions by displaying predictions in their biological context. Furthermore, WholeCellViz enables researchers to compare predictions within and across simulations by allowing users to simultaneously display multiple visualizations.

**Conclusion:**

WholeCellViz was designed to facilitate exploration, analysis, and communication of whole-cell model data. Taken together, WholeCellViz helps researchers use whole-cell model simulations to drive advances in biology and bioengineering.

## Background

Whole-cell computational models promise to predict how complex cellular behaviors such as growth and replication arise from individual molecules and their interactions. Recently, we developed the first whole-cell model of a single cell, the Gram-positive bacterium *Mycoplasma genitalium*[1]. The model predicts the dynamics of every molecular species over the entire cell cycle, accounting for the specific function of every annotated gene product. The model’s simulations produce rich data containing valuable insights into cellular behavior. For example, the model’s simulations have generated new insights into cell cycle regulation, energy usage, and gene essentiality [1].

However, the large number of whole-cell model predictions – over 50 billion data points in a typical dataset – makes directly analyzing the predictions time consuming and cumbersome. Furthermore, directly analyzing the model’s predictions requires deep knowledge of mathematical modeling, computer programming, and the unique data structures used to represent the model’s predictions.

Data visualization software is critically needed to help researchers realize the full potential of whole-cell models by enabling researchers to more quickly and efficiently analyze whole-cell model simulations. We developed WholeCellViz to enable researchers to easily visualize whole-cell model predictions. WholeCellViz provides researchers interactive animations as well as time series plots to easily explore whole-cell model predictions. Furthermore, WholeCellViz facilitates comparisons within and across simulations by enabling researchers to view grids of animations and plots.

Interactive data visualization is becoming increasingly important as biological data continues to grow in complexity and volume. Data visualization can help scientists identify subtle patterns in large data sets leading to important scientific findings. For example, Lum et al. used Iris to visualize genetic data from 272 breast cancer patients [2]. Iris revealed a specific genetic profile for women with low estrogen receptor expression, but high survival rates, a group which now receives targeted treatment for breast cancer. Shannon et al. used Cytoscape to visually link biomolecular networks with high-throughput data on various molecular states and functional annotations [3]. Baliga et al. used Cytoscape to obtain a systems-level understanding of *Halobacterium* energy transduction by visualizing its protein interaction network [4]. Pathway Tools enables researchers to visually integrate genomic, proteomic, and metabolomic data [5]. Chang et al. and Paley et al. used the Pathway Tools Omics Viewer to investigate the role of individual metabolic networks in bacterial infection [6,7]. MulteeSum was developed to visualize three-dimensional gene expression data, and has been used to gain insight into *Drosophila* development [8,9].

Here we describe WholeCellViz’s implementation, features, and visualizations. We also provide two examples of how WholeCellViz can be used to analyze whole-cell model predictions.

## Implementation

### Software overview

WholeCellViz is composed of a web-based front-end application and a back-end web server. The front-end displays visualizations to the user. The back-end server stores over 2 TB of simulation data using a combination of a MySQL relational database and JSON (JavaScript Object Notation) files, and sends this data to the front-end as requested by the user. WholeCellViz was developed as a web application in order to enable platform independence, simple installation, instant developer updates, and data streaming.

### Back-end storage server

Our whole-cell model software stores the predicted values of each biological variable at each time point using a set of MATLAB data files. We converted this data into the JSON format using custom Python scripts. We stored the metadata for each simulation, and the label and units for each data point in the database. The WholeCellViz front-end requests metadata and JSON file(s) from the back-end server as needed to display visualizations.

### Graphical user interface

The WholeCellViz front-end was implemented in HTML5 and JavaScript using the native canvas to maximize performance. We used JQuery (http://jquery.com) to implement event handling, animations, and AJAX calls.

The visualizations were implemented using an extensible framework designed to enable additional visualizations to be easily added to WholeCellViz. Specifically, each visualization extends a common class by defining methods for requesting and displaying data. The source code contains a template for constructing additional visualizations.

We developed the time series plots using the Flot (http://www.flotcharts.org) plotting library. We used the JQuery and JQuery UI (http://jqueryui.com) libraries to implement WholeCellViz’s grid layout and animation controls.

## Results and discussion

We developed WholeCellViz to accelerate data-driven discovery by visualizing whole-cell model simulation data. WholeCellViz uses simulation data to render 14 visualizations that display model predictions in their biological context. Time series plots supplement the visualizations by showing the detailed dynamics of one or multiple biological variables over time. WholeCellViz lays out these visualizations in an easily configurable grid. The animation timeline controls the simultaneous playback of all displayed animations in the grid. Hence, WholeCellViz is able to simultaneously visualize and animate multiple model predictions.

### Features

**Figure 1:**
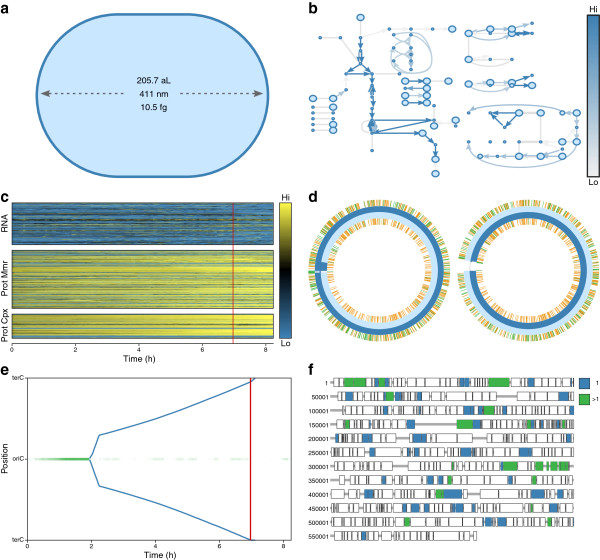
**Cell cycle dynamics view of one wild-type *in silico *cell at 7 h post-cell cycle initiation.** This view includes six animations which highlight the dynamics of the predicted metabolic fluxes and RNA and protein expression over the cell cycle. In particular, the view shows the onset of DNA replication, and the subsequent bidirectional movement of DNA polymerase on the chromosome. The view also highlights the onset of cytokinesis following the completion of DNA replication. **(a)** Instantaneous shape of *M. genitalium* as it initially elongates and later pinches at the septum, forming two daughter cells. **(b)** Metabolic map illustrating metabolite concentrations and reaction fluxes. Each metabolite is normalized to its mean concentration, and each reaction is normalized to its mean flux. Dark blue arrows indicate high reaction flux; light blue arrows indicate low reaction flux. Large circles indicate high metabolite concentrations; small circles indicate low metabolite concentrations. **(c)** Heatmap of the copy number of each RNA, protein monomer, and protein complex species. Each gene product is normalized to its mean copy number. Yellow indicates high expression; blue indicates low expression. **(d)** Instantaneous polymerization (blue), methylation (orange), strand break (red), and protein-binding status of the *M. genitalium* chromosomes. **(e)** Space-time plot illustrating the instantaneous chromosomal locations of the replication initiator DnaA and DNA polymerase. **(f)** Map of the protein-coding genes indicating protein synthesis. Each gene is colored according to the length of its longest nascent polypeptide. Green represents genes with one active ribosome; blue represents genes with multiple active ribosomes. An interactive version is available at http://wholecellviz.stanford.edu/cellCycle.

Figure 1 is a sample screenshot of WholeCellViz. We use this figure to describe the features of WholeCellViz.

#### Visualizations

**Figure 2:**
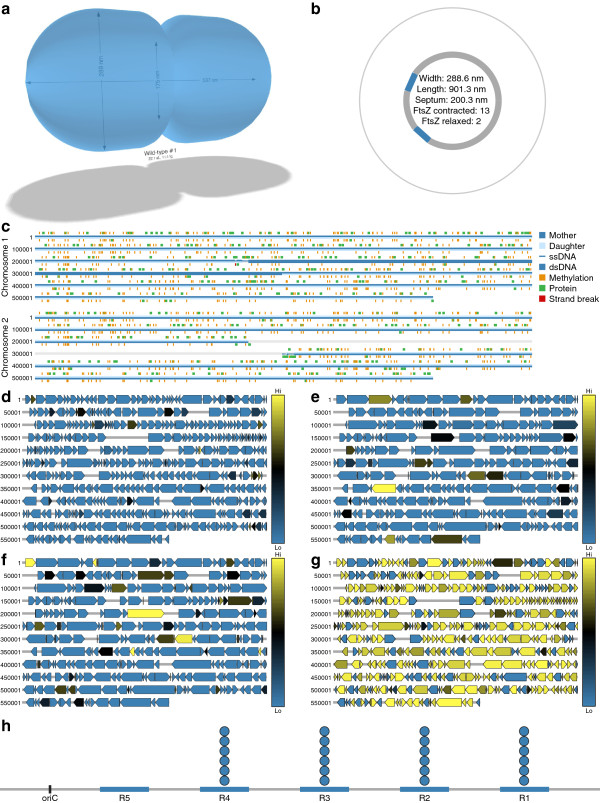
**Additional WholeCellViz visualizations.** Visualizations highlight one wild-type *in silico* cell at various time points. **(a)** Instantaneous shape of *M. genitalium* as it initially elongates and later pinches at the septum, forming two daughter cells. **(b)** Instantaneous FtsZ contractile ring size. FtsZ rings iteratively contract at the cell septum to pinch the cell membrane during cytokinesis. **(c)** Instantaneous polymerization (blue), methylation (orange), strand break (red), and protein-binding status of the *M. genitalium* chromosomes. **(d**–**g)** Heatmaps of the copy number dynamics of immature proteins **(d)**, immature RNA **(e)**, mature proteins **(f)**, and mature RNA **(g)**. Each gene product is normalized to its maximal expression. Yellow indicates high expression; blue indicates low expression. **(h)** Occupancy of the oriC functional DnaA boxes which recruit DNA polymerase to the oriC to initiate replication.

WholeCellViz contains 14 visualizations that animate specific model predictions within their biological context. These visualizations are listed in Table 1 and illustrated in Figures 1 and 2. Together, these 14 visualizations are capable of displaying 88% of the model’s predictions. These visualizations are also interactive. For example, hovering over the metabolism (Figure 1b) visualization reveals tooltips which display metabolite names, compartments, and concentrations. The gene expression panel’s tooltips display gene names, descriptions, and instantaneous copy numbers (Figure 1c). Clicking on a gene in the translation panel (Figure 1f) opens a new tab which displays the gene’s entry in the WholeCellKB model organism database [10].

**Table 1:** WholeCellViz visualizations

**Visualization**	**Figure**	**URL**
Cell shape	1a	http://wholecellviz.stanford.edu/CellShape
Cell shape (3D)	2a	http://wholecellviz.stanford.edu/CellShape3D
Chromosome (linear)	2c	http://wholecellviz.stanford.edu/Chromosome1
Chromosome (circular)	1d	http://wholecellviz.stanford.edu/Chromosome2
Chromosome (space-time)	1e	http://wholecellviz.stanford.edu/ChrSpaceTime
Cytokinesis	2b	http://wholecellviz.stanford.edu/Cytokinesis
Gene expression	1c	http://wholecellviz.stanford.edu/GeneExp
Immature protein expression	2d	http://wholecellviz.stanford.edu/NascentProtExp
Immature RNA expression	2e	http://wholecellviz.stanford.edu/NascentRnaExp
Metabolism	1b	http://wholecellviz.stanford.edu/Metabolism
Mature protein expression	2f	http://wholecellviz.stanford.edu/MatureProtExp
Mature RNA expression	2g	http://wholecellviz.stanford.edu/MatureRnaExp
Replication initiation	2h	http://wholecellviz.stanford.edu/RepInit
Translation	1f	http://wholecellviz.stanford.edu/Translation

#### Time series plots

**Figure 3:**
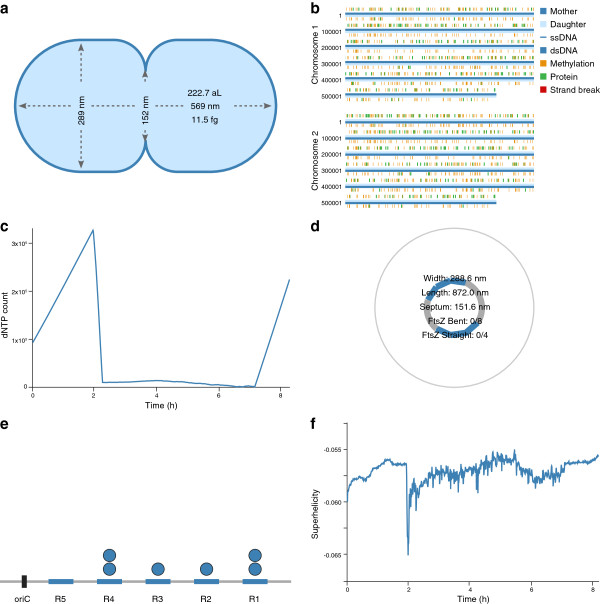
**Replication dynamics view of one wild-type *in silico *cell at 7.5 h post-cell cycle initiation. (a)** Instantaneous cell shape. **(b)** Instantaneous polymerization (blue), methylation (orange), strand break (red), and protein-binding status of the *M. genitalium* chromosomes. **(c)** Intracellular dNTP copy number dynamics. **(d)** Instantaneous FtsZ and cell septum sizes. **(e)** Instantaneous oriC DnaA box occupancy. **(f)** Superhelicity dynamics. An interactive version is available at http://wholecellviz.stanford.edu/replication.

WholeCellViz can also display line plots showing the values of one or multiple biological variables over time. For example, the middle-left panel of Figure 3 illustrates the temporal dynamics of the intracellular ATP copy number. Time series plots can also display the dynamics of biological variables across simulations, facilitating comparisons across simulations.

#### Animation timeline

The animation timeline at the bottom of the screen controls the simultaneous playback of all displayed visualizations. It provides play/pause, seek, speed, and repeat controls.

#### Layout editor

The layout editor is accessed by clicking the gear icon in the top-right corner of the visualization panels. The layout editor enables users to configure the grid dimensions and select the visualization or time series plot displayed in each panel.

#### Data import

Users can visualize data from any server running the server-side WholeCellViz software. The hosted version at http://wholecellviz.stanford.edu provides the over 3,000 described in Karr et al., 2012 [1]. Users can install the whole-cell model and WholeCellViz server software on their own machines, or use the whole-cell Linux virtual machine to execute and visualize new simulations. See below for more information about availability.

#### Graphical & data export

WholeCellViz exports the plotted data in JSON format and exports graphics in SVG format.

#### Data exploration using WholeCellViz

WholeCellViz can display multiple visualization panels to facilitate comparative and simultaneous analysis of multiple aspects of simulated cell physiology. In particular, WholeCellViz provides six preconfigured views to help users quickly get started. Each of the six views is a grid of visualizations selected to represent a particular aspect of cellular or population dynamics. These views enable users to explore hypotheses about the data. Here we discuss two case studies to illustrate the power of WholeCellViz to facilitate data exploration.

#### Replication dynamics

Figure 3 shows a screen shot of the replication dynamics view. This view displays several perspectives on DNA replication and cytokinesis: cell shape, chromosome dynamics, cytokinesis, replication initiation, and dNTP copy number. First, the view shows that before replication initiates the cell contains a single chromosome and steadily accumulates an increasingly large pool of dNTPs. Second, the view shows that once a sufficiently large oriC DNA complex forms, replication begins accompanied by a sharp drop in the dNTP level. Third, the view shows that replication then proceeds quickly until the dNTP supply is depleted, at which point the rate of replication slows. Finally, the view shows that the FtsZ ring contracts immediately following replication completion.

#### Population variance

**Figure 4:**
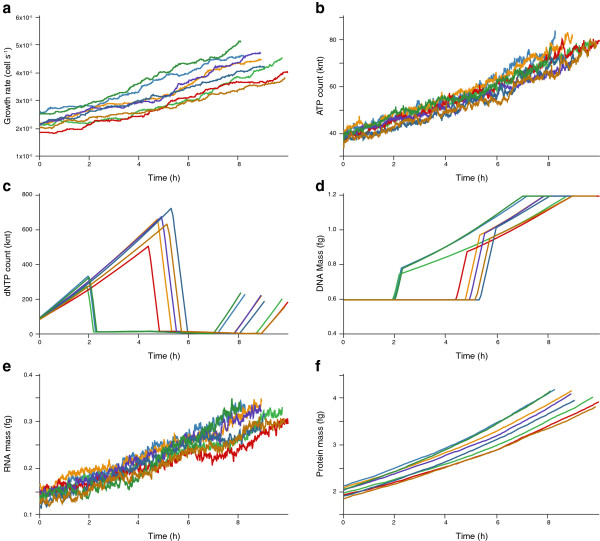
**Population variance view of eight wild-type *in silico *cells at 6 h post-cell cycle initiation.** View illustrates the temporal dynamics of the cellular growth rate **(a)**, ATP and total dNTP copy numbers **(b**, **c)**, and DNA, RNA, and protein masses **(d**–**f)**. Colors indicate the eight *in silico* cells. An interactive version is available at http://wholecellviz.stanford.edu/population.

Figure 4 shows a screen shot of the population variance view. This view presents summary statistics – growth rate, ATP copy number, dNTP copy number, DNA mass, RNA mass, and protein mass – for eight wild-type *in silico* cells. The view shows that the growth rate, ATP copy number, RNA mass, and protein mass have relatively little variance at the population level. The dNTP copy number and DNA mass have substantially more variance. In three simulations, the dNTP copy number is depleted more than two hours earlier than in the other simulations, and the DNA mass increases earlier in these simulations. This suggests that the timing of DNA replication initiation does not impact the cellular growth rate, ATP copy number, RNA mass, protein mass, or cell cycle length. Rather, the view suggests that metabolism is the primary factor controlling and coordinating the cell’s growth, chemical content, and division.

## Conclusions

WholeCellViz is a web-based program designed to facilitate exploration, and analysis of *in silico* biological experiments of whole-cell models. The software enables users to fully explore whole-cell model simulations, and displays whole-cell model predictions in their biological context using visualizations and time series plots. Furthermore, WholeCellViz’s grid layout feature enables users to display multiple visualizations and plots, enabling comparative analysis both within and across *in silico* cells.

Going forward, we plan to improve WholeCellViz as a tool for novel model analysis. We plan to develop new visualizations to communicate additional model predictions including DNA supercoiling and RNA and protein maturation. We also plan to develop enhanced plotting tools for detecting complex relationships among model predictions and analyzing stochastic variation. For example, scatter plots could be used to drill-down to specific time points and examine correlations among multiple variable in a single simulation, or among one variable across multiple simulations. Box plots could be used to compare the variance of variables across simulations.

To date only one whole-cell model has been developed. Consequently, we chose to focus WholeCellViz on the over 3,000 *M. genitalium* simulations described in Karr et al., 2012 [1]. Going forward, we plan to integrate WholeCellViz with other whole-cell models and simulation data servers as they become publicly available. Currently users can visualize alternative whole-cell model simulations by (1) running their own simulations using either our *M. genitalium* model or a similarly detailed model, (2) storing their simulations on their own server using the hybrid MySQL/JSON format described here, and (3) editing the back-end server URL configuration option from the WholeCellViz front-end. Researchers can achieve this either by installing the whole-cell model and WholeCellViz software on their own machine or by using our Linux virtual machine which contains both the whole-cell model and WholeCellViz software (see below for more information about availability). In the future, we also plan to enable researchers to configure and run whole-cell simulations through a simple graphical interface within WholeCellViz. However, this will require the development of more computationally efficient whole-cell model simulations.

Overall, whole-cell modeling is an emerging field that has the potential to accelerate the pace of biological discovery and enable rational bioengineering and personalized medicine. Data visualization software such as WholeCellViz is critically needed to help researchers access, explore, and analyze complex, high-dimensional whole-cell model simulations, as well as to accelerate model-driven biological discovery. With the current influx of big data in research and industry, WholeCellViz also serves as an example of how to use animation for scientific communication. We anticipate that WholeCellViz will play a critical role in realizing the full potential of whole-cell models.

## Availability and requirements

**Project name:** WholeCellViz

**Project home page:**http://wholecellviz.stanford.edu

**Operating system(s):** Platform independent

**Programming language:** HTML, JavaScript, PHP

**Other requirements:** Web browser

**License:** MIT license

**Any restrictions to use by non-academics:** None

WholeCellViz is available under the MIT license at http://wholecellviz.stanford.edu. The hosted version visualizes the over 3,000 simulations described in Karr et al., 2012 [1], and is also capable of visualizing simulations stored on other servers running the WholeCellViz server-side software. Researchers can install the whole-cell model and WholeCellViz software locally to execute and visualize new simulations. All source code is available open-source at SimTK: http://simtk.org/home/wholecell. A Linux virtual machine containing the whole-cell model and WholeCellViz server and client software is also available at SimTK.

## Abbreviations

AJAX: Asynchronous javascript and XML; ATP: Adenosine triphosphate; dNTP: Deoxynucleotide triphosphate; HTML: Hypertext markup language; JSON: Javascript object notation; oriC: Origin of replication; PHP: PHP: hypertext preprocessor; SVG: Scalable vector graphics; TB: Terabyte; URL: Uniform resource locator; XML: Extensible markup language.

## Competing interests

The authors declare that they have no competing interests.

## Authors’ contributions

RL and KR contributed equally to the conception and development of WholeCellViz. MC supervised the project. All authors wrote and approved the final manuscript.
